# Supplementary material for: Trio deep-sequencing does not reveal unexpected off-target and on-target mutations in Cas9-edited rhesus monkeys
Source: Nat Commun. 2019 Dec 4;10:5525. doi: 10.1038/s41467-019-13481-y (PMC6892871; doi:10.1038/s41467-019-13481-y)
Supplement: Supplementary file 1 — Supplementary Information [file 41467_2019_13481_MOESM1_ESM.pdf]

Supplementary figures and tables

Supplementary Fig. 1 | Generation of Cas9-mediated *MCPH1* gene knockout rhesus monkeys.

Supplementary Fig. 2 | Microscope image of Cas9-treated rhesus monkey embryos.

Supplementary Fig. 3 | Cas9-mediated *MCPH1* gene knockout in the cultured rhesus monkey embryos.

Supplementary Fig. 4 | Snapshot of the alignment of NGS reads within the target regions.

Supplementary Fig. 5 | The genetic relationship among the Cas9-treated monkeys.

Supplementary Fig. 6 | Genome-wide distribution of DNMs of the Cas9-edited monkeys.

Supplementary Fig. 7 | Venn diagram of the overlapping DNMs called using four different tools.

Supplementary Fig. 8 | Sanger validation results of rmO1^{ko} DNMs.

Supplementary Table 1 | Sample information.

Supplementary Table 2 | Quality control of whole genome sequencing data.

Supplementary Table 3 | Statistics of variants intersection rate between different tools.

Supplementary Table 4 | Summary of mutations located in the predicted off-target regions detected by WGS data.

Supplementary Table 5 | Oligonucleotides for generating sgRNA expression vectors.

Supplementary Table 6 | Primers for genotyping.

Supplementary Table 7 | Commands used for variant calling.

Supplementary Table 8 | Commands used for SNP filtering.

Supplementary Table 9 | Commands used for INDEL filtering.

Supplementary Table 10 | Commands for universal mask (UM)

Supplementary Fig. 1 | Generation of Cas9-mediated *MCPH1* gene knockout rhesus monkeys.

a, The schematic overview of sgRNA design targeting rhesus monkey *MCPH1* gene; **b,** photograph of two Ca9-edited rhesus monkeys (rmO3^{ko} and rmO4^{ko*}). **c,** *MCPH1* sequences carrying indels within the target regions of Cas9-edited rhesus monkeys. The PAM sequences are underlined and highlighted in red and the targeted sequences are in blue. The deletion sizes and ratios are indicated in parentheses.

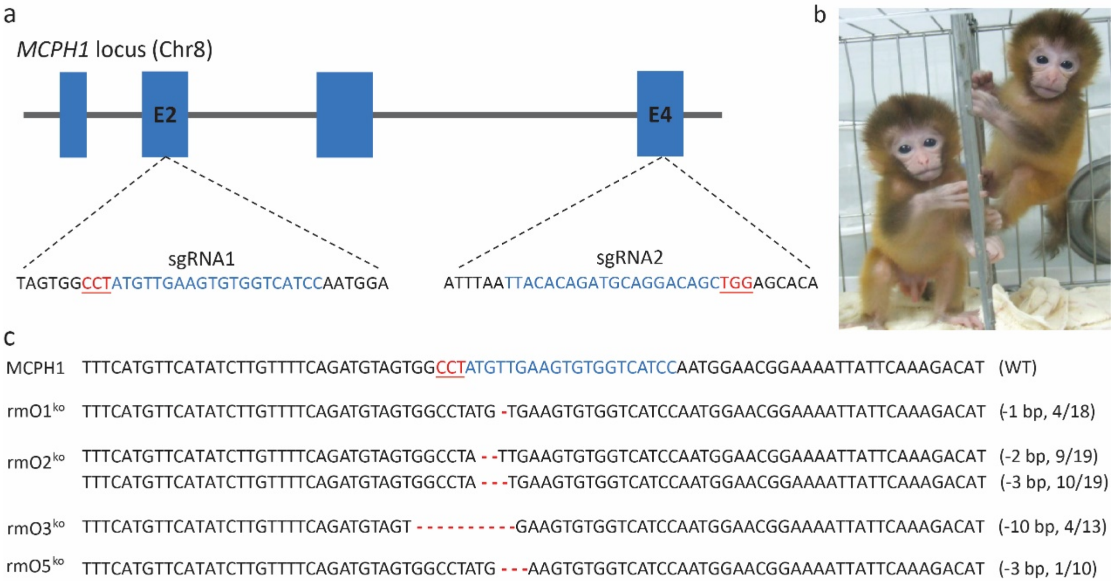

Supplementary Fig. 2 | Microscope image of Cas9-treated rhesus monkey embryos.

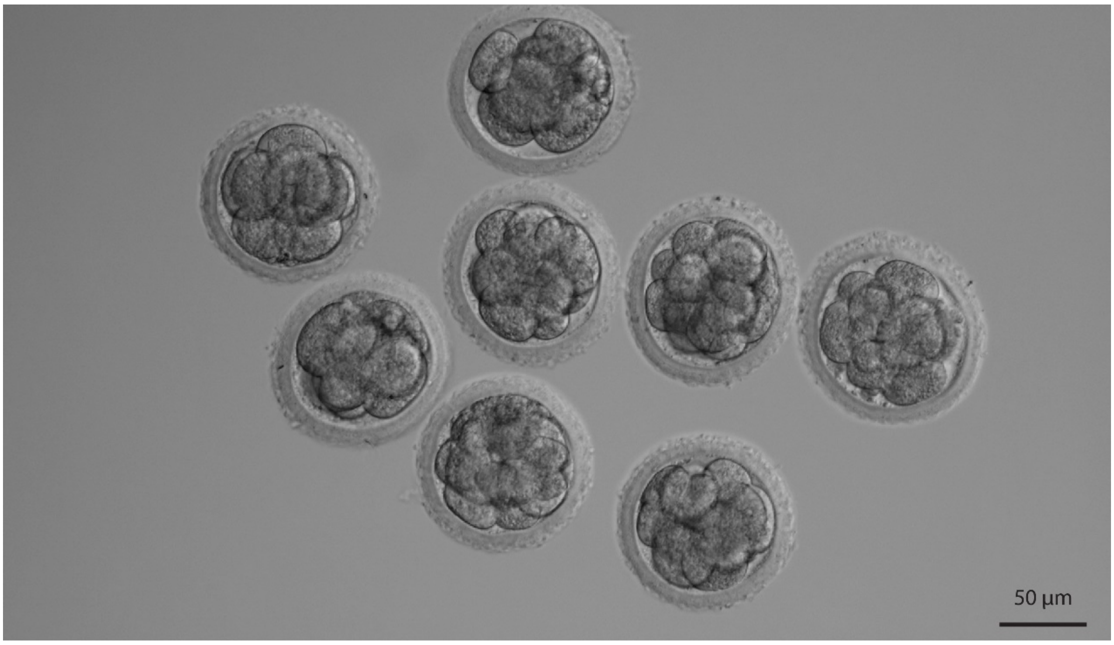

Supplementary Fig. 3 | Cas9-mediated *MCPH1* gene knockout in the cultured rhesus monkey embryos. The PAM sequences are underlined and highlighted in red; The targeted sequences are in green and the mutations in blue; deletions (-), and insertions (+). The size and ratios of deletions are indicated in parenthesis.

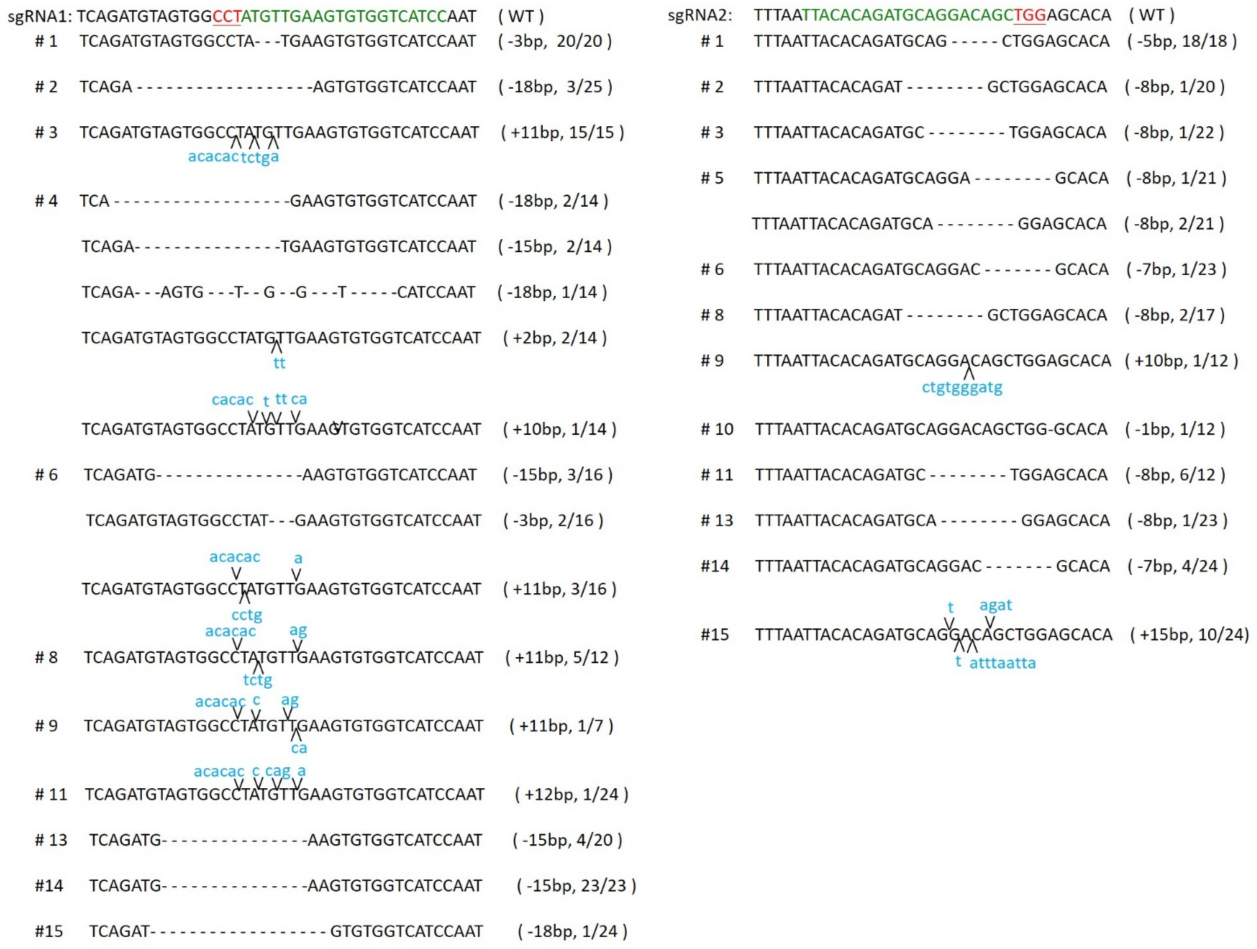

Supplementary Fig. 4 | Snapshot of the alignment of NGS reads within the target regions. The gray-highlighted region refers to sgRNA and "*" refers to deletion.

rmO2-B^{ko} sgRNA1

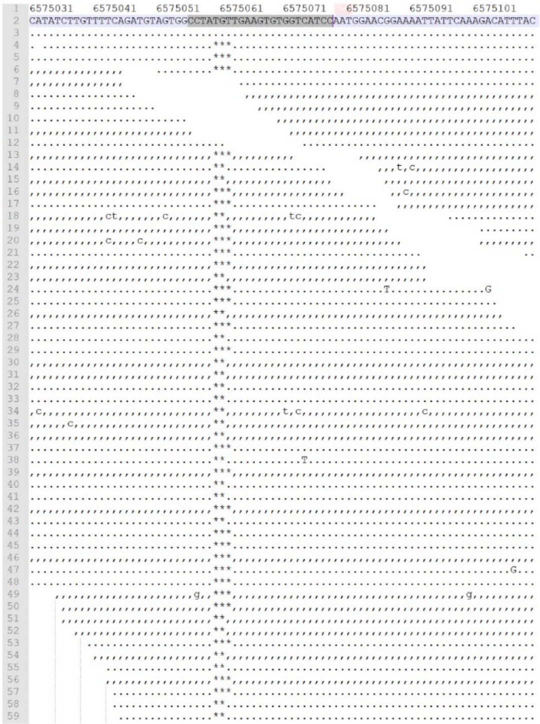

rmO2-B^{ko} sgRNA2

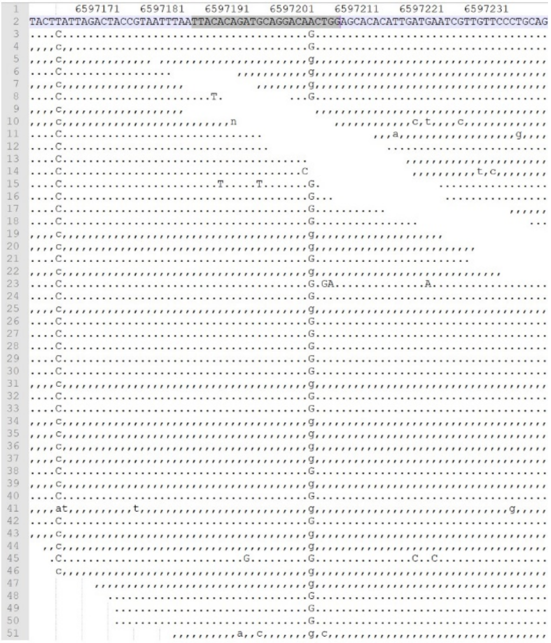

rmO2-L^{ko} sgRNA1

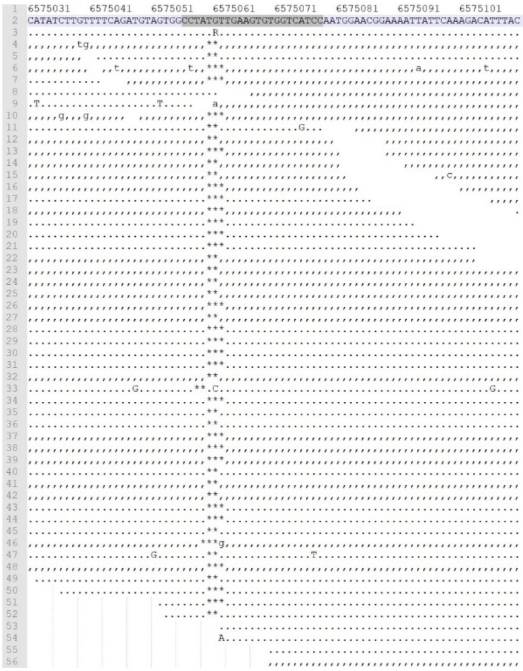

rmO2-L^{ko} sgRNA2

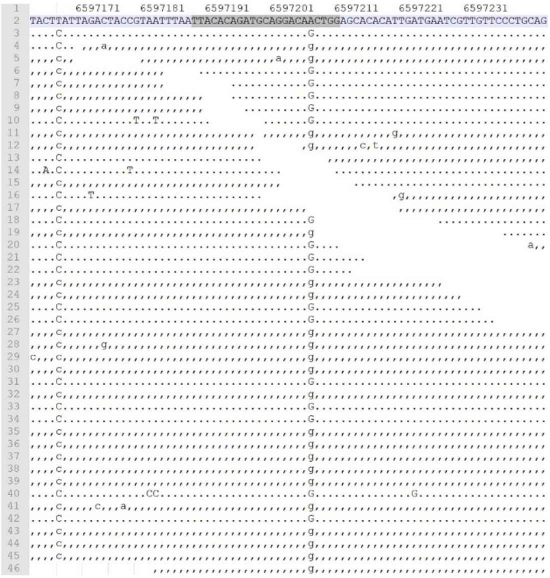

rmO2-M^{ko} sgRNA1

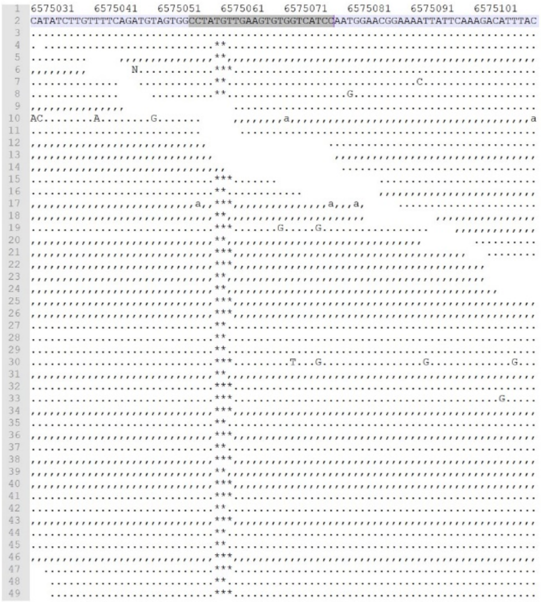

rmO2-M^{ko} sgRNA2

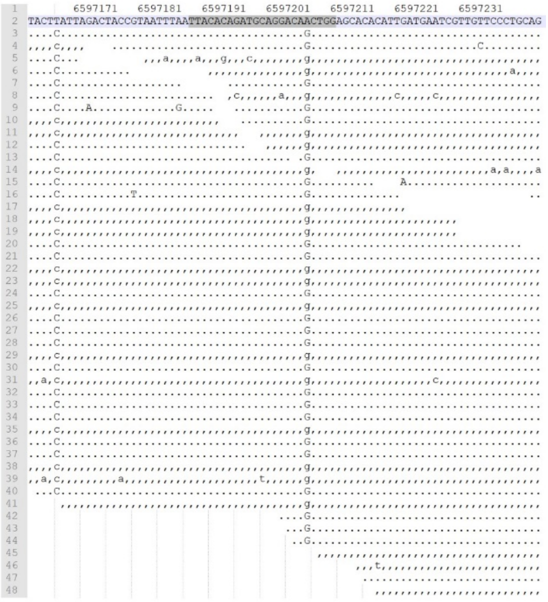

rmO1^{ko} sgRNA1

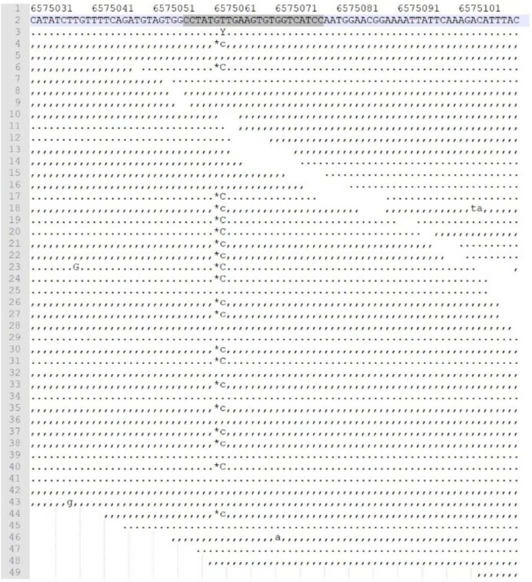

rmO1^{ko} sgRNA2

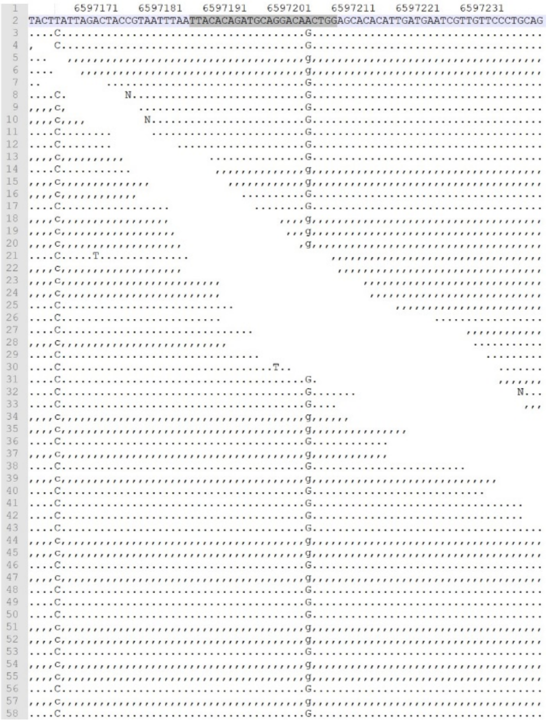

rmO3^{ko} sgRNA1

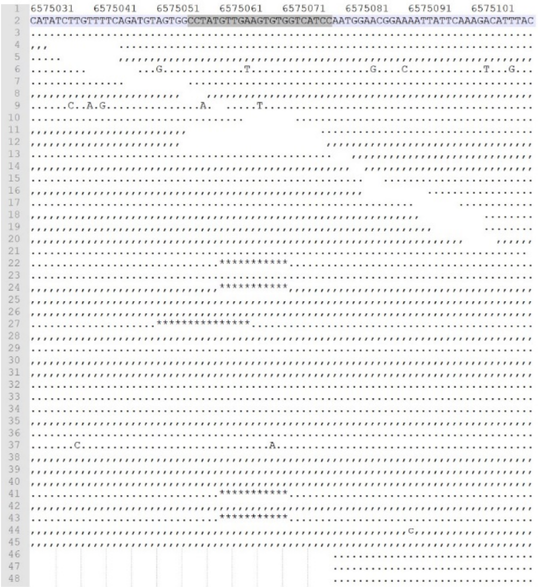

rmO3^{ko} sgRNA2

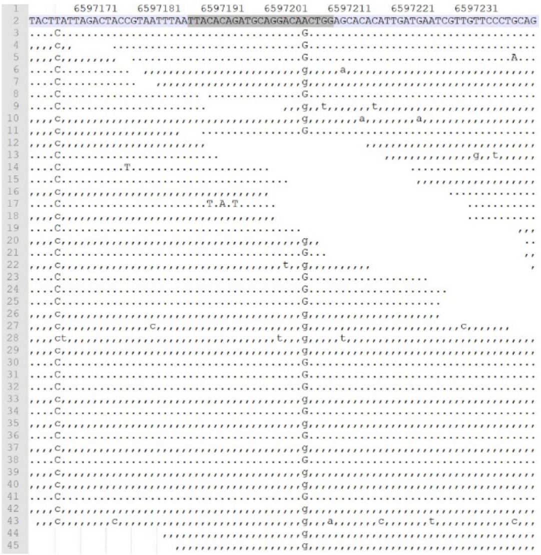

rmO4^{ko*} sgRNA1

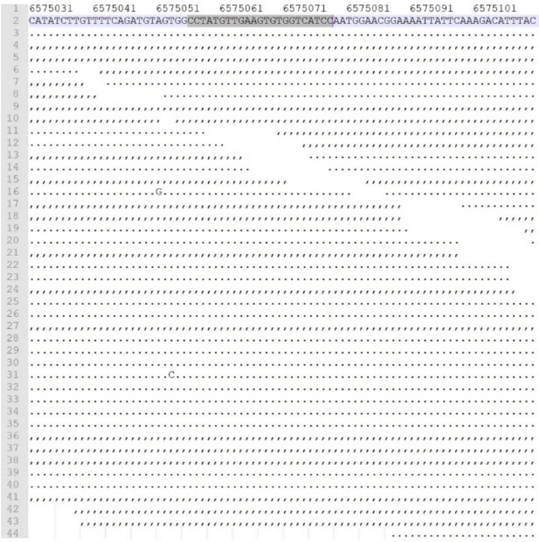

rmO4^{ko*} sgRNA2

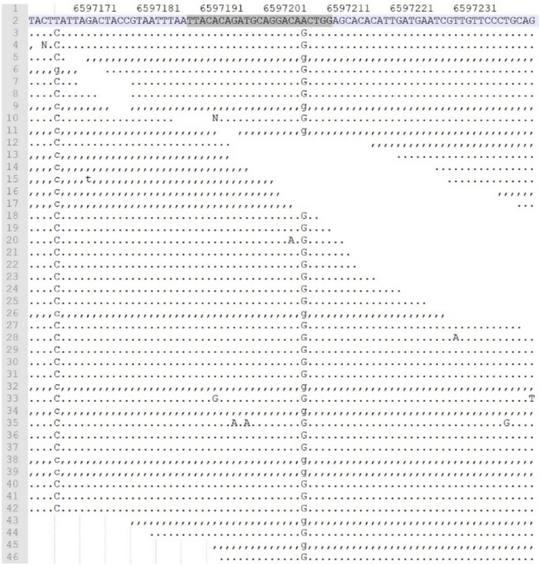

rmO5^{ko} sgRNA1

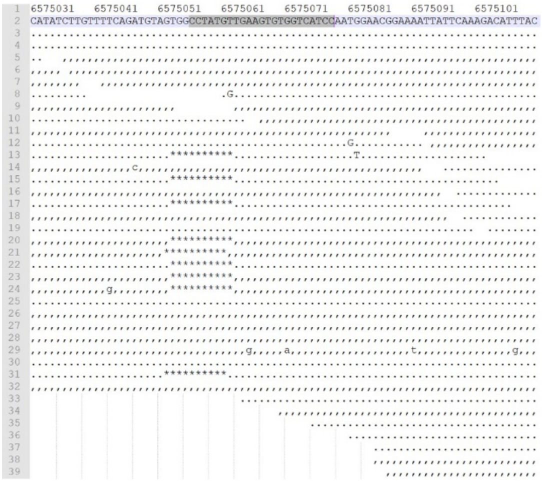

rmO5^{ko} sgRNA2

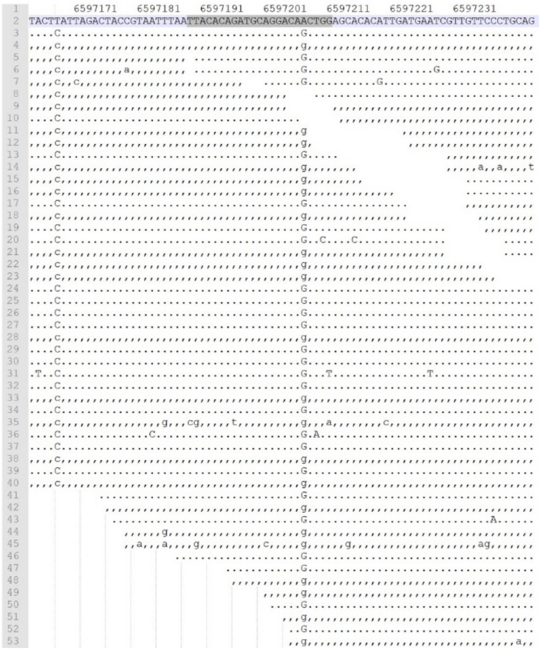

Supplementary Fig. 5 | The genetic relationship among the Cas9-treated monkeys. Each cell indicated an IBD score calculated by individual pair. The darker a cell is, the higher is the IBD score and a closer relationship.

Rhesus monkey family-1

	rmF^{wt}	rmM1^{wt}	rmO1^{ko}	rmO2-B^{ko}	rmO2-M^{ko}	rmO2-L^{ko}
rmF^{wt}	1					
rmM1^{wt}	0	1				
rmO1^{ko}	0.5	0.5	1			
rmO2-B^{ko}	0.4704	0.5	0.423	1		
rmO2-M^{ko}	0.4705	0.5	0.4224	0.9248	1	
rmO2-L^{ko}	0.4705	0.5	0.4224	0.9245	0.9245	1

Rhesus monkey family-2

	rmF^{wt}	rmM2^{wt}	rmO3^{ko}	rmO4^{wt*}	rmO5^{ko}
rmF^{wt}	1				
rmM2^{wt}	0	1			
rmO3^{ko}	0.5	0.5	1		
rmO4^{wt*}	0.5	0.5	0.4607	1	
rmO5^{ko}	0.5	0.5	0.4827	0.473	1

Cynomolgus monkey family

	cmF^{wt}	cmM^{wt}	cmO^{ki}
cmF^{wt}	1		
cmM^{wt}	0	1	
cmO^{ki}	0.5	0.5	1

Supplementary Fig. 6 | Genome-wide distribution of DNMs of the Cas9-edited monkeys. The blue and red triangles indicate the locations of the SNPs and INDELs respectively in the genomes.

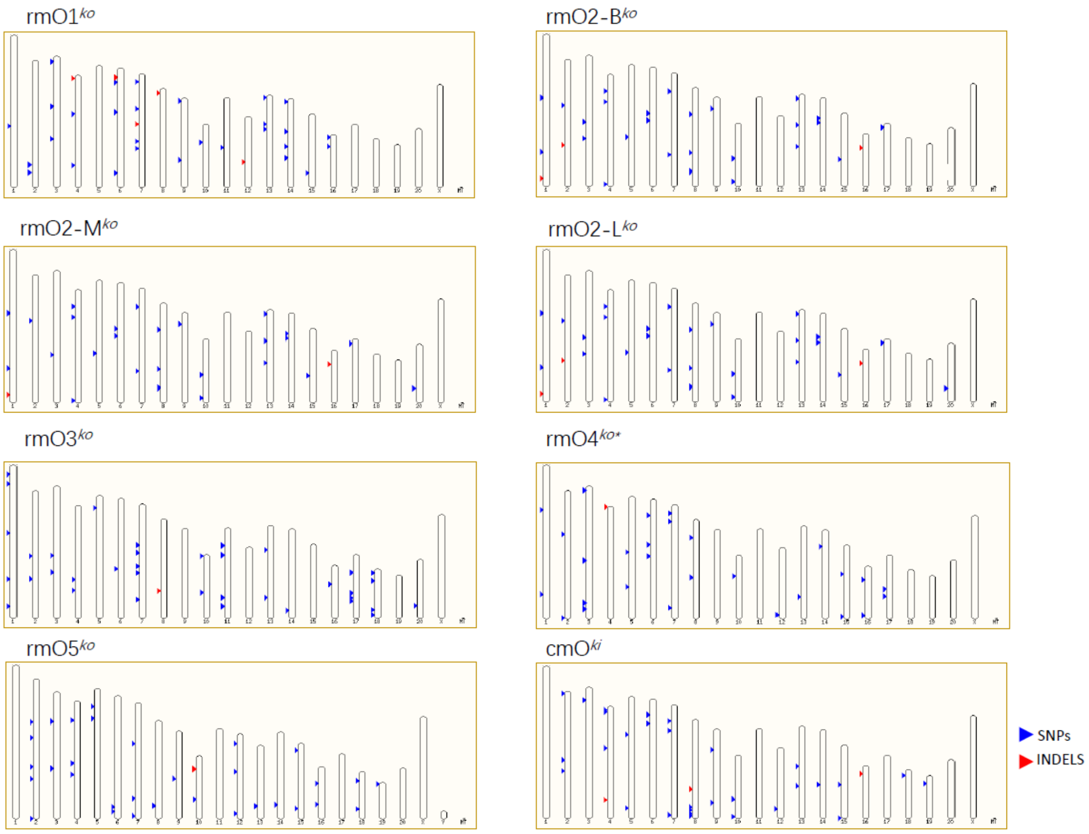

Supplementary Fig. 7 | Venn diagram of the overlapping DNMs called using four different tools.

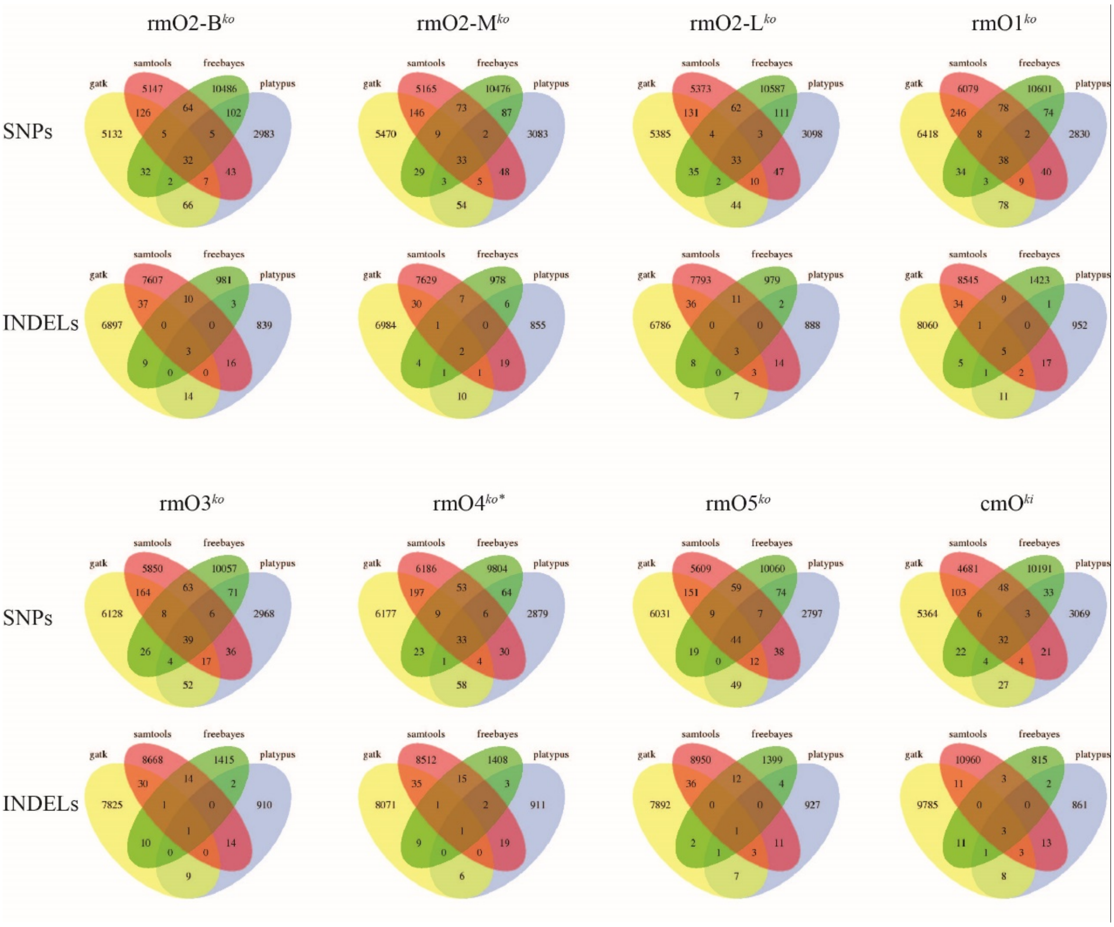

Supplementary Fig. 8 | Sanger validation results of rmO1^{ko} DNMs. F: father, M: mother, O: offspring. The red dashed box indicated SNPs and the red dotted line indicates indels.

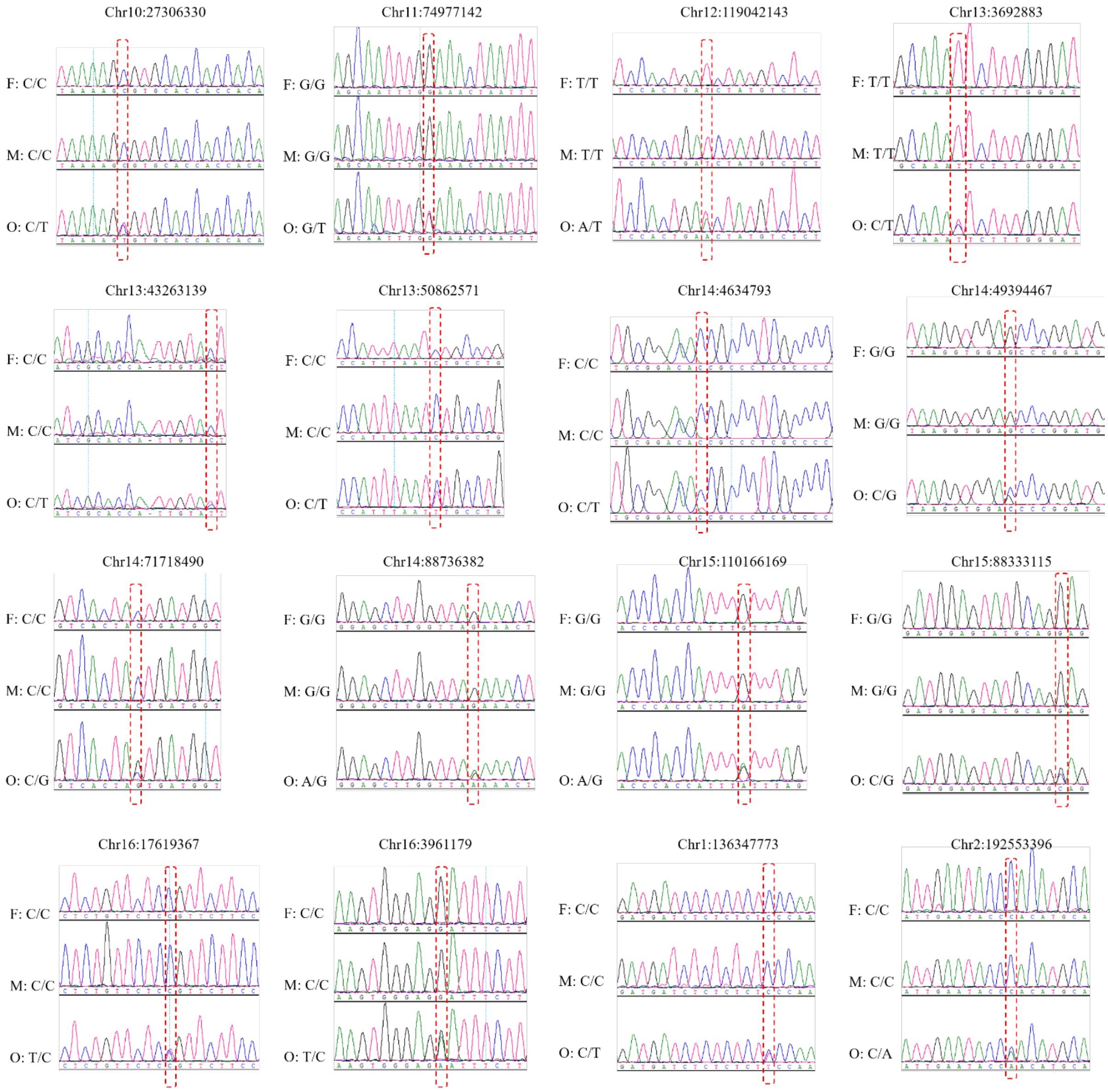

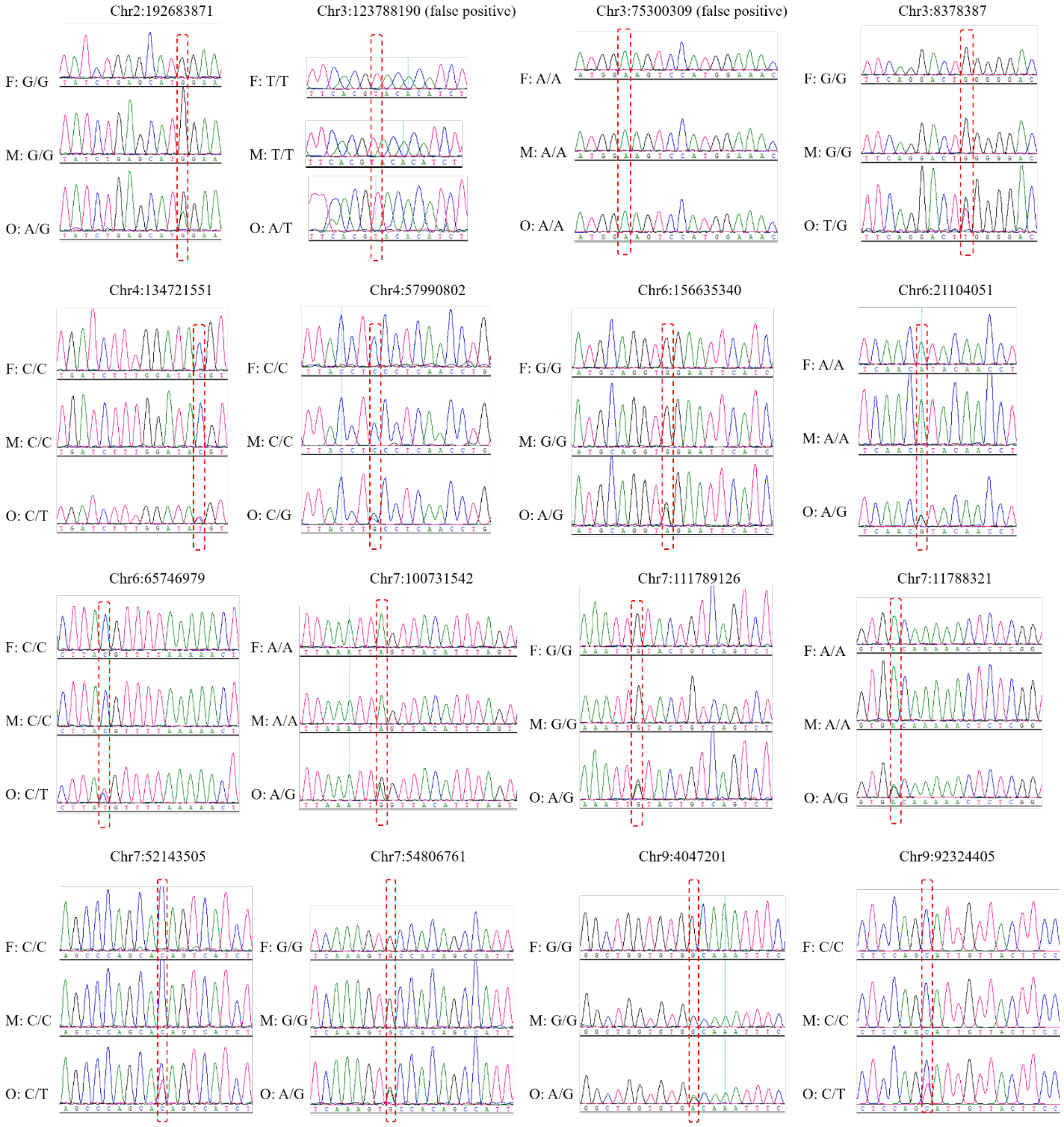

Chr2:192683871
F: G/G
M: G/G
O: A/G
Chr3:123788190 (false positive)
F: T/T
M: T/T
O: A/T
Chr3:75300309 (false positive)
F: A/A
M: A/A
O: A/A
Chr3:8378387
F: G/G
M: G/G
O: T/G
Chr4:134721551
F: C/C
M: C/C
O: C/T
Chr4:57990802
F: C/C
M: C/C
O: C/G
Chr6:156635340
F: G/G
M: G/G
O: A/G
Chr6:21104051
F: A/A
M: A/A
O: A/G
Chr6:65746979
F: C/C
M: C/C
O: C/T
Chr7:100731542
F: A/A
M: A/A
O: A/G
Chr7:111789126
F: G/G
M: G/G
O: A/G
Chr7:11788321
F: A/A
M: A/A
O: A/G
Chr7:52143505
F: C/C
M: C/C
O: C/T
Chr7:54806761
F: G/G
M: G/G
O: A/G
Chr9:4047201
F: G/G
M: G/G
O: A/G
Chr9:92324405
F: C/C
M: C/C
O: C/T

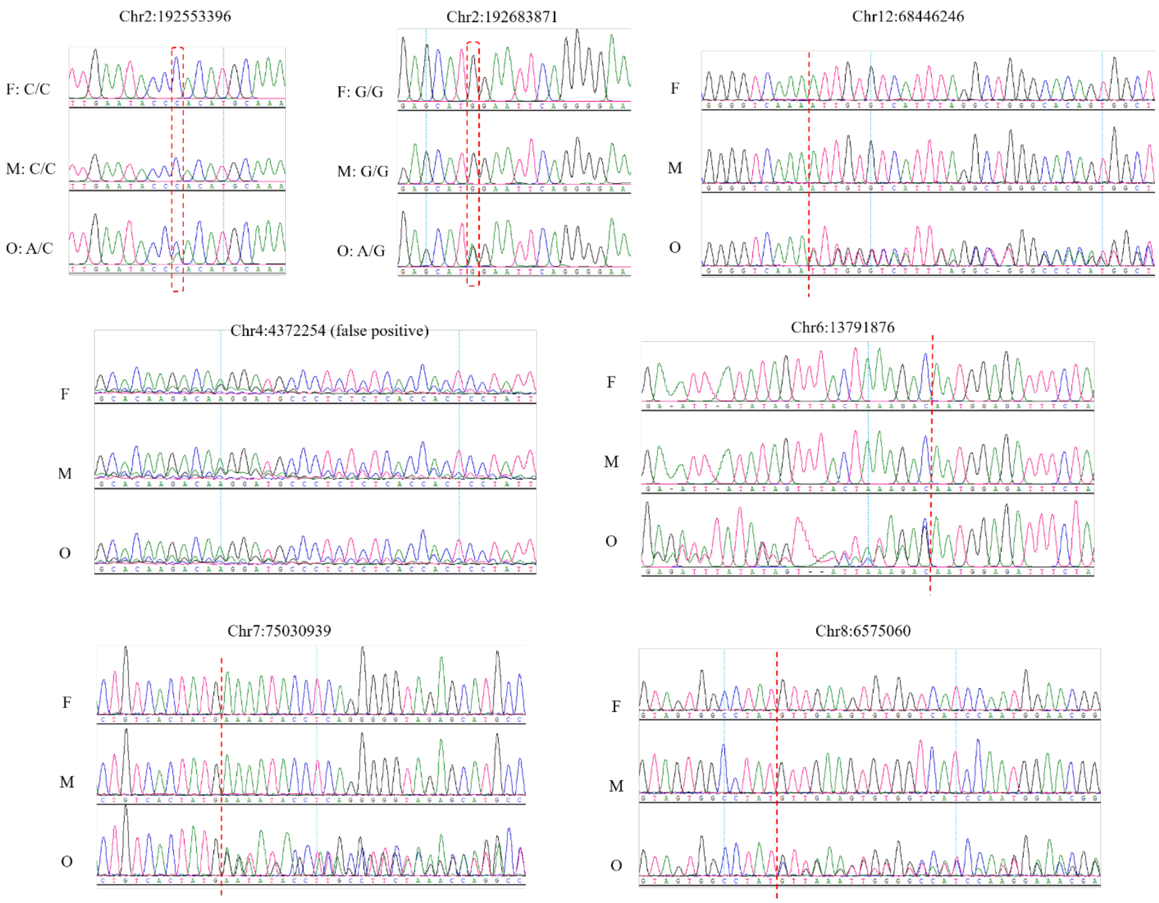

Chr2:192553396
F: C/C
M: C/C
O: A/C
Chr2:192683871
F: G/G
M: G/G
O: A/G
Chr12:68446246
F
M
O
Chr4:4372254 (false positive)
F
M
O
Chr6:13791876
F
M
O
Chr7:75030939
F
M
O
Chr8:6575060
F
M
O

Supplementary Table 1 | Sample information.

Father	Mother	Offspring	Sex	Date of Birth	Gestational day	Method of delivery	Genotyping	Status
rmF^{wt}	$rmM1^{wt}$	$rmO1^{ko}$	female	2015-05-28	138	premature	*MCPH1-/+*	live
		$rmO2^{ko}$	male	2015-05-28	138	premature	*MCPH1-/-*	dead
	$rmM2^{wt}$	$rmO3^{ko}$	male	2015-06-24	160	C-section	*MCPH1-/+*	live
		$rmO4^{ko*}$	female	2015-06-24	160	C-section	*MCPH1+/+*	live
		$rmO5^{ko}$	female	2015-06-24	160	C-section	*MCPH1-/+*	live

Supplementary Table 2 | Quality control of whole genome sequencing data.

Cluster	ID	Reads(R1)	Reads(R2)	Q30percent (R1)	Q30percent (R2)	meanQ30 percentage	Raw Depth	eDP mean	Duplication (%)	Mapped rate (%)	Properly Paired(%)	IS mean
10cas9	rmF^{wt}	437754192	437754192	91.4	80.96	86.18	45.81	40.1	12.82	0.99	96.15	403.42
10cas9	rmM1^{wt}	441379369	441379369	91.86	82.26	87.06	46.23	40.57	12.62	0.99	96.19	413.4
10cas9	rmM2^{wt}	444856449	444856449	92.13	82.04	87.08	45.23	41.01	12.37	0.99	96.24	413.56
10cas9	rmO1^{ko}	460430614	460430614	93.97	86.93	90.45	48.39	41.9	13.74	0.99	96.22	408.92
10cas9	rmO2-B^{ko}	438596862	438596862	91.81	79.77	85.79	45.98	41.5	10.93	0.99	96.33	418.2
10cas9	rmO2-M^{ko}	427690705	427690705	91.28	80.44	85.86	44.85	40.53	9.83	0.99	96.36	418.51
10cas9	rmO2-L^{ko}	432868473	432868473	91.58	80.27	85.93	45.35	40.31	11.42	0.99	96.1	417.96
10cas9	rmO3^{ko}	445885275	445885275	93.49	85.75	89.62	46.84	41.39	11.96	0.99	96.24	406.18
10cas9	rmO4^{ko*}	451706794	451706794	93.73	85.85	89.79	45.92	41.68	12.49	0.99	96.24	413.87
10cas9	rmO5^{ko}	453838377	453838377	93.71	84.61	89.16	47.6	41.96	12.17	0.99	96.14	417.99
rm-average		443500711	443500711	92.5	82.89	87.69	46.22	41.1	12.04	0.99	96.22	413.2
3fas	cmF^{wt}	613554604	613554604	95.47	89.88	92.67	64.08	55.38	15.82	1	96.09	398.42
3fas	cmM^{wt}	718249285	718249285	95.26	89.15	92.2	76.11	64.56	15.31	0.99	96.04	397.13
3fas	cmO^{ki}	606545633	606545633	95.47	89.74	92.61	68.42	55.56	14.67	1	96.19	396.88
cm-average		646116507	646116507	95.4	89.59	92.5	69.54	58.5	15.27	0.99	96.11	397.48

Note: eDP: effective read depth; IS: insert size

Supplementary Table 3 | Statistics of variants overlapping rate between different tools.

Type		SNPs								INDELs							
Trio		rmF^{wt},<br>rmM1^{wt},<br>rmO2-B^{ko}	rmF^{wt},<br>rmM1^{wt},<br>rmO2-M^{ko}	rmF^{wt},<br>rmM1^{wt},<br>rmO2-L^{ko}	rmF^{wt},<br>rmM1^{wt},<br>rmO1^{ko}	rmF^{wt},<br>rmM1^{wt},<br>rmO3^{ko}	rmF^{wt},<br>rmM1^{wt},<br>rmO4^{ko}*	rmF^{wt},<br>rmM1^{wt},<br>rmO5^{ko}	cmF^{wt},<br>cmM1^{wt},<br>cmO^{ki}	rmF^{wt},<br>rmM1^{wt},<br>rmO2-B^{ko}	rmF^{wt},<br>rmM1^{wt},<br>rmO2-M^{ko}	rmF^{wt},<br>rmM1^{wt},<br>rmO2-L^{ko}	rmF^{wt},<br>rmM1^{wt},<br>rmO1^{ko}	rmF^{wt},<br>rmM1^{wt},<br>rmO3^{ko}	rmF^{wt},<br>rmM1^{wt},<br>rmO4^{ko}*	rmF^{wt},<br>rmM1^{wt},<br>rmO5^{ko}	cmF^{wt},<br>cmM1^{wt},<br>cmO^{ki}
Freebayes		14764912	14749570	14736766	14252445	14543314	14387107	14552891	17616002	2387828	2383757	2384310	2396452	2381389	2349867	2379194	2543011
GATK		18018008	17557248	16756111	18032040	17902620	17903588	17900994	20886574	4357146	4354284	4355881	4374952	4351204	4352004	4350673	3903592
Samtools		14493928	14488592	14487188	14472446	14305092	13296260	14301298	17046056	4106000	4088103	4082281	4023035	4044644	4037729	4050129	4675619
Platypus		16629543	16629435	16630154	16662612	15765770	16553433	16556609	19246854	2195249	2193336	2193079	2236743	2218780	2223224	2223854	2100526
Variants intersection	Freebayes:GATK	13721038	13351797	12731502	13278615	13546578	13416121	13559050	16564340	1399652	1396679	1396534	1404035	1394766	1380007	1395202	1527891
	Freebayes:Samtools	13454580	13444091	13446691	13038119	13281921	12214276	13280131	15975769	1270662	1269124	1268457	1272065	1263930	1250890	1264964	1422145
	Freebayes:Platypus	13398805	13381003	13373090	12945539	12582760	13086159	13222029	15979509	1240242	1237363	1236826	1250419	1241344	1228391	1241705	1354743
	GATK: Samtools	13368497	13012704	12417176	13378470	13223716	12298689	13224557	16047367	1662639	1661152	1661594	1670566	1662048	1663487	1664362	1743692
	GATK: Platypus	15513505	15103492	14411226	15566418	14721570	15464302	15465080	18205000	1787313	1785047	1785333	1819084	1806280	1809208	1809155	1760124
	Samtools: Platypus	13237726	13230774	13229364	13242257	12473172	12180259	13096441	15797183	1490712	1488542	1488696	1508507	1499673	1500740	1501174	1561970
	Freebayes:GATK: Samtools	12981622	12636105	12056932	12607967	12840368	11818725	12842144	15606509	1189273	1187555	1187142	1191771	1183481	1172218	1184816	1344031
	Freebayes: GATK: Platypus	1301504	1266230	1207104	1259490	1223708	1273371	1286201	1567464	1182299	1179716	1179203	1192471	1183806	1172169	1184427	1303130
	Freebayes: Samtools: Platypus	12816209	12806050	12808035	12436133	12065384	11661886	12670917	15334800	1090881	1088525	1088057	1099085	1091035	1080614	1091889	1235646
	GATK: Samtools: Platypus	12790948	12451626	11878275	12812498	12063389	11784938	12670704	15429328	1400120	1398327	1398625	1416895	1408579	1409751	1410135	1476287
	Freebayes: GATK: Samtools: Platypus	12508536	12176530	11615962	12157438	11791952	11403992	12386275	15099333	1062339	1060092	1059767	1070284	1062359	1052582	1063453	1205860
Max intersection rate		0.863	0.840	0.802	0.840	0.824	0.858	0.866	0.886	0.484	0.483	0.483	0.479	0.479	0.473	0.478	0.574
Min intersection rate		0.694	0.694	0.693	0.674	0.659	0.637	0.692	0.723	0.244	0.243	0.243	0.245	0.244	0.242	0.244	0.309

Supplementary Table 4 | Summary of mutations located in the predicted off-target regions detected by WGS data.

sgRNA	No. of mismatches	SpeedSeq		Cas-Offinder		No. of off-target sites
		No. of NGG genomic sites	No. of NAG genomic sites	No. of NGG genomic sites	No. of NAG genomic sites	
sgRNA1	0	1	0	1	0	0
	1	2	0	0	0	0
	2	2	1	0	0	0
	3	4	9	4	24	0
	4	51	102	96	323	0
	5	687	1,034	1,010	2,634	0
	6	-	-	8,574	14,722	0
	7	-	-	59,847	66,295	0
sgRNA2	0	0	0	0	0	0
	1	1	0	1	0	0
	2	3	0	1	0	0
	3	14	11	17	17	0
	4	129	149	205	231	0
	5	1,179	1,428	1,526	1,874	0
	6	-	-	10,612	13,633	0
	7	-	-	58,734	77,563	0

Supplementary Table 5 | Oligonucleotides for generating sgRNA expression vectors.

**Oligo**	**Sequence (5’ to 3’)**
MCPH1 sgRNA1 T7 Top	TAGGGGATGACCACACTTCAACAT
MCPH1 sgRNA1 T7 Bottom	AAACATGTTGAAGTGTGGTCATCC
MCPH1 sgRNA2 T7 Top	TAGGTTACACAGATGCAGGACAGC
MCPH1 sgRNA2 T7 Bottom	AAACGCTGTCCTGCATCTGTGTAA

Supplementary Table 6 | Primers for genotyping.

Primer	Sequence (5’ to 3’)
MCPH1 exon2 PCR-F	CTGGTCTCAAATCCCTGACTTCGTG
MCPH1 exon2 PCR-R	TCTCCCACCACTTACCCATTGCTTT
MCPH1 exon4 PCR-F	TTTGGGAAGTTTGATTTATACTGAC
MCPH1 exon4 PCR-R	GAAGAAGTCATAGGATAAAATAATC

Supplementary Table 7 | Commands used for variant calling.

Algorithm	Version	Command line
BWA-MEM	0.7.12-r1039	bwa mem ref.fa read1.fq read2.fq
GATK HaplotypeCaller	3.6	java -jar GenomeAnalysisTK.jar -T HaplotypeCaller -R ref.fa -I dedup.bam --emitRefConfidence GVCF -o output.gvcf
Platypus	0.8.1	Platypus callVariants –bamFiles=dedup.bam –refFile=ref.fa --maxVariants=20 --maxReads=30000000
Samtools	1.3.1	Samtools mpileup -go 10macaque.by120.bcf -uf ref.fa dedup.bam
Freebayes	v1.0.2-15-g357f175	Freebayes -f ref.fa dedup.bam
Delly	v0.7.7	delly call -t sv_type -g ref.fa dedup.bam -o sv_type.bcf; bcftools view sv_type.bcf >sv_type.vcf
Pindel	V0.2.5b9	pindel -i bam.config.txt -f ref.fa -o sv_type

Supplementary Table 8 | Commands used for SNP filtering.

<table>
<tr><td>VCF sets</td><td>SF</td><td>GF</td><td>UM</td></tr>
<tr><td>GATK</td><td>java -jar GenomeAnalysisTK.jar \
-T VariantFiltration \
-R ref.fa \
-V VariantCalls.SNP.vcf \
--filterExpression "QD < 2.0 || FS > 60.0 || MQ < 30.0 || MQRankSum < -12.5 || ReadPosRankSum < -8.0 " \
--filterName "my_snp_filter" \
-o VariantCalls.filtered.SNP.vcf</td><td>python genotype_filter.py "DP:15-100" "GQ>30" VariantCalls.filtered.vcf.gz</td><td>vcftools --gzvcf SNPs.vcf.gz --exclude-bed UM_bedfile --recode --recode-INFO-all --stdout</td></tr>
<tr><td>Platypus</td><td>vcffilter -f "SbPval >0.001 & HapScore <13 & MQ >30 & QD >2 & NR >0 & NF >0" VariantCalls.SNP.vcf.gz</td><td>python genotype_filter.py "GQ>30" VariantCalls.filtered.vcf.gz</td><td>vcftools --gzvcf SNPs.vcf.gz --exclude-bed UM_bedfile --recode --recode-INFO-all --stdout</td></tr>
<tr><td>Freebayes</td><td>vcffilter -f "QUAL >30 & QUAL / AO >10 & SAF >0 & SAR >0 & RPL >1 & RPR >1 & MQM >30 & MQMR >30" VariantCalls.SNP.vcf.gz</td><td>python genotype_filter.py "DP:15-100" "GQ>30" VariantCalls.filtered.vcf.gz</td><td>vcftools --gzvcf SNPs.vcf.gz --exclude-bed UM_bedfile --recode --recode-INFO-all --stdout</td></tr>
<tr><td>Samtools</td><td>vcffilter -f "MQ >30 & RPB >0.001 & MQB >0.001 & BQB >0.001 & MQSB >0.001" VariantCalls.SNP.vcf.gz</td><td>-</td><td>vcftools --gzvcf SNPs.vcf.gz --exclude-bed UM_bedfile --recode --recode-INFO-all --stdout</td></tr>
</table>

Supplementary Table 9 | Commands used for INDEL filtering.

VCF sets	SF	GF	UM
GATK	java -jar GenomeAnalysisTK.jar \ -T VariantFiltration \ -R ref.fa \ -V VariantCalls_chr$i.INDELS.vcf \ --filterExpression "QD < 2.0 || FS > 200.0 || ReadPosRankSum < -20.0" \ --filterName "my_indel_filter" \ -o VariantCalls.filtered.INDELs.vcf	python genotype_filter.py "GQ>30" VariantCalls.filtered.vcf.gz	vcftools --gzvcf INDELs.vcf.gz --exclude-bed UM_bedfile --recode --recode-INFO-all --stdout
Platypus	vcffilter -f "SbPval >0.001 & HapScore <13 & MQ >30 & QD >2 & NR >0 & NF >0" VariantCalls.INDELS.vcf.gz	python genotype_filter.py "GQ>30" VariantCalls.filtered.vcf.gz	vcftools --gzvcf INDELs.vcf.gz --exclude-bed UM_bedfile --recode --recode-INFO-all --stdout
Freebayes	vcffilter -f "QUAL >30 & QUAL / AO >10 & SAF >0 & SAR >0 & RPL >1 & RPR >1 & MQM >30 & MQMR >30" VariantCalls.INDELS.vcf.	python genotype_filter.py "GQ>30" VariantCalls.filtered.vcf.gz	vcftools --gzvcf INDELs.vcf.gz --exclude-bed UM_bedfile --recode --recode-INFO-all --stdout
Samtools	vcffilter -f "MQ >30 & MQSB >0.001" VariantCalls_chr$i.INDELS.vcf.gz | bgzip > hardfilter.INDELS.vcf.gz	-	vcftools --gzvcf INDELs.vcf.gz --exclude-bed UM_bedfile --recode --recode-INFO-all --stdout

Supplementary Table 10 | Commands for universal mask (UM)

<table>
<tr><td rowspan="5">Mappability mask</td><td>splitfa ref.fa   150 | gzip > read91.fastq.gz</td></tr>
<tr><td>bwa aln -t 12 -R 1000000 -O 3 -E 3 ref.fa   read91.fastq.gz > read91.aln.sai</td></tr>
<tr><td>bwa samse ref.fa read91.aln.sai read91.fastq.gz | gzip > read91.aln.sam.gz</td></tr>
<tr><td>gzip -dc read91.aln.sam.gz | gen_raw_mask.pl > rawMask_91_aln.fa</td></tr>
<tr><td>gen_mask -l 91 -r 0.5 rawMask_91_aln.fa > mask_91_50.fa</td></tr>
<tr><td>LCR mask</td><td>mdust ref.fa -w 7 -v 28 -c | gzip > mdust.mask.txt.gz</td></tr>
</table>
