# Supplementary material for: Trio deep-sequencing does not reveal unexpected off-target and on-target mutations in Cas9-edited rhesus monkeys
Source: Nat Commun. 2019 Dec 4;10:5525. doi: 10.1038/s41467-019-13481-y (PMC6892871; doi:10.1038/s41467-019-13481-y)
Supplement: Supplementary file 6 — Description of Additional Supplementary Files [file 41467_2019_13481_MOESM6_ESM.pdf]

Title: Supplementary Data 1
Description: Summary of DNMs and LFDMs detected in the Cas9-treated monkeys.

Title: Supplementary Data 2
Description: Statistics of SNPs and INDELs in each filter step.
